# Aducanumab: Appropriate Use Recommendations

**DOI:** 10.14283/jpad.2021.41

**Published:** 2021-07-20

**Authors:** Jeffrey Cummings, P. Aisen, L.G. Apostolova, A. Atri, S. Salloway, M. Weiner

**Affiliations:** 1 Chambers-Grundy Center for Transformative Neuroscience, Department of Brain Health, School of Integrated Health Sciences, University of Nevada Las Vegas (UNLV), Las Vegas, NV, USA; 2 Alzheimer's Treatment Research Institute, University of Southern California, San Diego, CA, USA; 3 Departments of Neurology, Radiology, Medical and Molecular Genetics, Indiana University School of Medicine, Indianapolis, Indiana, USA; 4 Banner Sun Health Research Institute, Banner Health, Sun City, AZ, USA; 5 Center for Brain/Mind Medicine, Harvard Medical School, Boston, MA, USA; 6 Butler Hospital and Warren Alpert Medical School of Brown University, Providence, RI, USA; 7 Departments of Radiology and Biomedical Imaging, Medicine, Psychiatry and Neurology, University of California San Francisco, San Francisco, CA, USA; 8 1380 Opal Valley Street, 89052, Henderson, NV, USA

**Keywords:** Alzheimer's disease, aducanumab, Aduhelm™, appropriate use, titration, ARIA, amyloid imaging, MRI

## Abstract

Aducanumab has been approved by the US Food and Drug Administration for treatment of Alzheimer's disease (AD). Clinicians require guidance on the appropriate use of this new therapy. An Expert Panel was assembled to construct Appropriate Use Recommendations based on the participant populations, conduct of the pivotal trials of aducanumab, updated Prescribing Information, and expert consensus. Aducanumab is an amyloid-targeting monoclonal antibody delivered by monthly intravenous infusions. The pivotal trials included patients with early AD (mild cognitive impairment due to AD and mild AD dementia) who had confirmed brain amyloid using amyloid positron tomography. The Expert Panel recommends that use of aducanumab be restricted to this population in which efficacy and safety have been studied. Aducanumab is titrated to a dose of 10 mg/kg over a 6-month period. The Expert Panel recommends that the aducanumab be titrated to the highest dose to maximize the opportunity for efficacy. Aducanumab can substantially increase the incidence of amyloid-related imaging abnormalities (ARIA) with brain effusion or hemorrhage. Dose interruption or treatment discontinuation is recommended for symptomatic ARIA and for moderate-severe ARIA. The Expert Panel recommends MRIs prior to initiating therapy, during the titration of the drug, and at any time the patient has symptoms suggestive of ARIA. Recommendations are made for measures less cumbersome than those used in trials for the assessment of effectiveness in the practice setting. The Expert Panel emphasized the critical importance of engaging in a process of patient-centered informed decision-making that includes comprehensive discussions and clear communication with the patient and care partner regarding the requirements for therapy, the expected outcome of therapy, potential risks and side effects, and the required safety monitoring, as well as uncertainties regarding individual responses and benefits.

**A**ducanumab (Aduhelm™) has been approved by the US Food and Drug Administration (FDA) for the treatment of Alzheimer's disease (AD). The Prescribing Information for aducanumab (1) provides key facts on aducanumab such as dose, titration, pharmacokinetics, and side effects. The Clinical Studies section describes the clinical trials that led to the approval of aducanumab. Many details of the clinical use of this new agent are not detailed in the Prescribing Information (1) and there is a need for specific recommendations regarding how to use aducanumab appropriately. Experts with experience in AD research, AD clinical trials and drug development, AD clinical care, and use of aducanumab were assembled to develop consensus recommendations for the appropriate use of aducanumab in clinical practice.

The Prescribing Information (1) provides the “on label” prescribing instructions. The Expert Panel recommends that the appropriate use of aducanumab in real-world clinical practice should pragmatically mimic the use of aducanumab in the EMERGE and ENGAGE clinical trials that led the FDA to approve aducanumab. After the initial Prescribing Information was published, the FDA adjusted the indication section from “indicated for the treatment of Alzheimer's disease” to “indicated for the treatment of Alzheimer's disease…should be initiated in patients with mild cognitive impairment or mild dementia stage of the disease, the population in which treatment was initiated in clinical trials” (1, 2). Some of the Expert Panel recommendations are more specific or more restrictive than the information provided in the Prescribing Information (1). The recommendations are within the scope of use articulated in the Prescribing Information (1) The Expert Panel describes the appropriate use of aducanumab for the practicing clinician; we do not address trial outcomes, approval strategies, cost, insurance coverage, or reimbursement issues. The Expert Panel recommendations apply to practices in the Unites States where aducanumab is currently approved. Recommendations may change as more data on the use of aducanumab and more data from the trials become available. These recommendations are meant to assist practitioners in using aducanumab safely; they do not replace clinician judgement in the delivery of care to individual patients.

## Overview

Aducanumab is a monoclonal antibody directed to the N-terminus of the amyloid beta peptide (Aß). It was derived through a process of reverse translation in which blood lymphocytes from healthy elderly individuals who were cognitively normal or had unusually slow cognitive decline served as a source of antibody genes for the generation of recombinant human antibodies (3).

The Expert Panel recommends that patients treated with aducanumab closely resemble those included in the pivotal clinical trials (4, 5). Pragmatic adjustments will be required for use of aducanumab outside of the trial setting, and the translation of clinical trial protocol requirements to clinical practice is summarized in Table 1. Efficacy and safety have been assessed in the early AD population of patients with mild cognitive impairment (MCI) due to AD and mild dementia due to AD confirmed by amyloid positron emission tomography (PET) and are unknown for individuals with preclinical AD, those with more severe AD dementia, or those with cognitive impairment that is not confirmed to be AD by Aß studies.

**Table 1:** Clinical trial enrollment criteria and appropriate use criteria for aducanumab in clinical practice

Participant Feature	Clinical Trial Enrollment Criteria	Appropriate Use in Clinical Practice
Age	50–85	Younger or older patients meeting all other criteria for treatment could be considered candidates for aducanumab
Diagnosis	Clinical criteria for MCI due to AD or mild AD dementia	Clinical criteria for MCI due to AD or mild AD dementia
Scale scores at baseline	CDR Global Score 0.5; MMSE 24–30; RBANS Delayed Memory Score of 85 or less	MMSE 21–30 or equivalent such as MoCA 17–30
Amyloid status	Amyloid positive PET (visual read)	Amyloid positive PET (visual read) or CSF findings consistent with AD
Genetic testing	Consent for APOE genotyping	Genotyping should be discussed with the patient/care partner. ARIA risk should be described, and the patient's preferences assessed.
Neurological examination	Non-AD neurological disorders, stroke, and TIA excluded	Non-AD neurological disorders excluded
Cardiovascular history	Angina; myocardial infarction; congestive heart failure excluded	Stable cardiovascular conditions required; clinical decision can be exercised on the ability of the patient to participate safely with the therapeutic regimen
Medical history	Excluded: clinically significant systemic illness; diabetes than cannot be managed; uncontrolled hypertension (systolic > 165; diastolic > 100); history of cancer unless in remission for 5 years or localized to skin or prostate; impaired liver function; hepatitis; HIV infection	Stable medical conditions required; clinical decision can be exercised on the ability of the patient to participate safely with the therapeutic regimen
Psychiatric history	Unstable psychiatric illness in the past 6 months; alcohol or substance abuse in the past year; use of cannabinoids; positive urine tests for excluded substances	Must be stable psychiatrically; clinical decision can be exercised on the ability of the patient to participate safely with the therapeutic regimen
Reproductive status	Female subjects who are pregnant or breast feeding excluded; female subjects who are of childbearing age must be practicing contraception	Female subjects who are pregnant or breast feeding excluded; female subjects who are of childbearing age must be practicing contraception
Clotting status	Bleeding disorders, anticoagulants excluded	Patients on anticoagulants are excluded
Concomitant medications	Cholinesterase inhibitors and memantine allowed	Patients can be on standard of care with cholinesterase inhibitors and memantine
Baseline MRI	Baseline MRI finding that excluded participation: acute or subacute hemorrhage, macrohemorrhage, greater than 4 microhemorrhages, cortical infarction (>1.5 cm), 1 lacunar infarction (>1.5 cm), superficial siderosis, or diffuse white matter disease	Patients should be excluded if there is evidence of acute or subacute hemorrhage, macrohemorrhage, greater than 4 microhemorrhages, cortical infarction (>1.5 cm), 1 lacunar infarction (>1.5 cm), > 1 area of superficial siderosis, or diffuse white matter disease
Care support	Reliable informant or care partner	May be living independently or with a care partner
Informed consent	Must be signed by participant and care partner	Patient and care partner must understand the nature and requirements of therapy (e.g, monthly infusions to be performed indefinitely) and the expected outcome of therapy (slowing of decline of clinical features)


Aß — amyloid beta protein; AD — Alzheimer's disease; APOE — apolipoprotein E; CDR — Clinical Dementia Rating; cm — centimeter; CSF — cerebrospinal fluid; HIV — human immunodeficiency virus; MMSE — Mini Mental State Examination; MoCA — Montreal Cognitive Assessment; MRI — magnetic resonance imaging; PET — positron emission tomography; RBANS — Repeatable Battery for the Assessment of Neuropsychological Status; TIA — transient ischemic attack

### Appropriate Patient

#### Diagnosis

The Expert Panel recommends that patients appropriate for treatment with aducanumab have a diagnosis of early AD established by a diagnostic evaluation that includes: 1) detailed history that is sufficient to establish the nature and time course of cognitive symptoms, functional changes, and behavioral status; 2) objective corroboration of cognitive decline using standardized testing; 3) detailed neurological and physical examination; 4) review of all current medications and supplements; 5) laboratory testing sufficient to exclude other concomitant disorders that can cause cognitive decline including a complete blood count, electrolyte panel, thyroid stimulating hormone, lipids and triglycerides, liver function tests, and serum vitamin B12 level; and 6) magnetic resonance imaging (MRI) of the brain to rule out other conditions that could present with cognitive decline (e.g., normal pressure hydrocephalus, vascular dementia, slow going neoplasm, subdural hematoma) and to assess possible exclusions for use of aducanumab (discussed below) (6, 7, 8). This assessment will determine if the patient has clinical findings consistent with early AD.

Patients with early AD meet the clinical criteria of stage 3 and 4 of the FDA staging approach (9). Stage 3 consists of individuals with subtle or more apparent detectable abnormalities on sensitive neuropsychological measures and mild but detectable functional impairment. The functional impairment in this stage is not severe enough to warrant a diagnosis of overt dementia. Stage 4 includes individuals with cognitive impairment and mild but definite functional decline.

To quantify the cognitive and functional changes, early AD patients in the aducanumab trials had scores on the Clinical Dementia Rating (CDR) (10) global rating of 0.5. This instrument assesses cognitive (memory, orientation, judgment, and problem solving) and functional (community affairs, home and hobbies, and personal care) domains. In addition, trial participants had Mini Mental State Examination (MMSE) (11) scores of 24–30. The MMSE is commonly used in clinical practice and is a useful tool for identifying appropriate patients. The standard error of measurement on the MMSE is 1 point, and the minimum detectable difference is 3 points (12, 13). The test-retest reliability of MMSE is 2–4 points (14). These studies indicate that scores of 21 and higher would not be detectably different from the range of MMSE scores of patients included in the pivotal trials (MMSE range of 24–30). The Phase 1B study of aducanumab had encouraging results in patients with MMSE scores of 20–30 (15). The Expert Panel recommends that patients with MMSE scores of 21 or higher or who have a similar level of performance on an alternate reliable and valid assessment are appropriate for treatment with aducanumab. An alternative assessment that provides reliable information similar to that of the MMSE is the Montreal Cognitive Assessment (MoCA) (16). The MoCA is a more challenging test than the MMSE resulting in lower scores when compared to the MMSE. Scores of 17 and higher on the MoCA are equivalent to MMSE scores of 21–30 in early symptomatic AD (17). In settings where neuropsychological testing is available, a diagnosis of early AD can be based on more extensive cognitive, functional, and behavioral assessments (18).

#### Use of cognitive enhancing agents in aducanumab candidates

A newly diagnosed patient with MCI due to AD may be started on aducanumab since cholinesterase inhibitors and memantine are not approved for this stage of AD. Patients with early AD may be on a cholinesterase inhibitor or memantine when referred for possible treatment with aducanumab; these patients can remain on their standard of care while being treated with aducanumab. Patients diagnosed with mild AD dementia can have treatment with aducanumab before or following initiation of treatment with a cholinesterase inhibitor. If patients with MCI progress to mild AD dementia, treatment with a cholinesterase inhibitor (donepezil, rivastigmine, galantamine) can be considered. Memantine is not approved for mild AD dementia. If patients progress to moderate or severe AD, memantine treatment can be considered as monotherapy or in conjunction with a cholinesterase inhibitor (19).

#### Amyloid status

All patients included in the pivotal trials had positive amyloid positron emission tomography (PET). Demonstration of amyloid burden is critical to establishing the presence of the target for amyloid lowering therapies. The clinical diagnosis of AD is often not confirmed by amyloid studies and up to 40% of patients diagnosed with early AD do not have the amyloid pathology when studied with amyloid imaging (20). Appropriate Use Criteria of amyloid imaging suggest that the imaging is appropriate when: a) there is a cognitive complaint and cognitive impairment has been objectively confirmed impairment; b) AD is a possible diagnosis, but the diagnosis is uncertain after a comprehensive evaluation by a dementia expert; and c) knowledge of the presence or absence of amyloid-beta pathology is expected to increase diagnostic certainty and alter management (21). These criteria are fulfilled in the situation where a patient is being considered for treatment with aducanumab: they have the symptoms of early AD, additional diagnostic certainty is needed, and management will be based on the outcome.

Three amyloid PET tracers are approved by the FDA: florbetapir, florbetaben, and flutametamol (22, 23, 24). Table 2 provides the criteria for a positive scan for each tracer. Scan interpretation is best done by radiologists or nuclear medicine specialists; training programs for amyloid PET interpretation are available for each ligand. The Expert Panel recommends that programs offering aducanumab treatment and using amyloid PET to confirm the diagnosis of AD should ensure the availability of individuals properly trained in amyloid PET interpretation.

**Table 2:** Criteria for a positive amyloid PET for the three approved amyloid PET tracers (from drugs@FDA: FDA-Approved Drugs)

PET Tracer	Criteria for Interpreting as a Positive Scan
Florbetapir (Amyvid™)	A positive scan will have either: Two or more brain areas (each larger than a single cortical gyrus) in which there is reduced or absent gray-white contrast; OR, one or more areas in which gray matter radioactivity is intense and clearly exceeds radioactivity in adjacent white matter.
Florbetaben (Neuraceq™)	β-amyloid positive — smaller area(s) of tracer uptake equal to or higher than that present in white matter extending beyond the white matter rim to the outer cortical margin involving the majority of the slices within at least one of the four brain regions (“moderate” β-amyloid deposition), or a large confluent area of tracer uptake equal to or higher than that present in white matter extending beyond the white matter rim to the outer cortical margin and involving the entire region including the majority of slices within at least one of the four brain regions.
Flutemetamol (Vizamyl™)	Positive scans show at least one cortical region with reduction or loss of the normally distinct grey-white matter contrast. These scans have one or more regions with increased cortical grey matter signal (above 50–60% peak intensity) and/or reduced (or absent) grey- white matter contrast (white matter sulcal pattern is less distinct). A positive scan may have one or more regions in which grey matter radioactivity is as intense or exceeds the intensity in adjacent white matter.

Lumbar puncture and assessment of cerebrospinal fluid (CSF) biomarkers (Aβ42, Aβ40 total tau, phosphorylated tau [p-tau]) provide an alternative to amyloid PET and are more widely available (25). Several CSF measures can be indicative of the presence of AD including low Aβ42, low Aβ42/Aβ40 ratio, abnormal Aβ42/tau ratios, and abnormal Aβ42/p-tau ratios (26, 27, 28). Practitioners should use Clinical Laboratory Improvement Amendments (CLIA)-certified facilities and follow the laboratory's guidelines for optimal AD-related assays. If CSF results are ambiguous, amyloid imaging is recommended. Amyloid PET and CSF AD signature studies provide equally valid information (29); CSF Aβ42 levels correlate inversely with brain amyloid on PET with CSF levels declining as Aβ is deposited in the cortex (30). Changes in CSF Aβ42 levels precede changes in amyloid PET (31); individuals with abnormal CSF and normal amyloid PET imaging are usually without symptoms and they lack evidence of amyloid plaques which are the target of aducanumab. The Expert Panel recommends that these patients not be treated with aducanumab. Re-imaging with amyloid PET in 1–3 years may be warranted in this group of individuals.

Lumbar puncture can be performed by physicians, nurse practitioners, or physicians' assistants/associates with low patient morbidity and high safety (32). Lumbar puncture may not be possible in those with pathological or surgical changes of the lumbar spine; fluoroscopic guidance may be useful in such cases. Lumbar puncture is contraindicated in those with clotting disorders or who are on anticoagulants. Prothrombin time (PT) and partial thromboplastin time (PTT) can be obtained to ensure normal clotting parameters before proceeding with lumbar puncture.

Amyloid imaging or CSF biomarker analyses in persons with the clinical features of early AD will reveal that some of these cognitively impaired individuals do not have AD, exhibit evidence of neurodegeneration, and fulfill criteria for suspected non-Alzheimer pathology (SNAP) (33). Discovery of the non-amyloid status of these individuals assists clinicians in making management decisions (34). The Expert Panel recommends that individuals with SNAP not be treated with aducanumab.

Lumbar puncture with findings consistent with AD or PET with elevated brain amyloid confirm the diagnosis of AD in patients with the clinical syndrome of early AD. Failure to confirm the diagnosis of AD with amyloid biomarkers could result in administering aducanumab to patients who do not have AD and who lack the target pathology of the agent. The Expert Panel recommends that all patients considered for treatment with aducanumab have the diagnosis of AD confirmed by clinically validated amyloid studies such as amyloid PET or CSF analysis.

#### Genetic testing

Genetic testing to determine the apolipoprotein E (APOE) genotype of the participants was required in the pivotal trials. ARIA of the effusion (ARIA-E) or hemorrhagic (ARIA-H) type are more common in APOE *ε* 4 (APOE-4) gene carriers and understanding this effect in trials is important (35). ARIA may be more common in APOE-4 homozygotes and can be severe (36). The Prescribing Information (1) instructions for use of aducanumab do not require APOE genotyping and the dosing and monitoring of individuals with and without an APOE-4 allele are identical. The Expert Panel recommends that patients and care partners be engaged in a patient-centered discussion of the risk that an APOE-4 genotype confers for the risk of ARIA. This discussion will determine if genotype information would influence their decision to be treated with aducanumab and if they wish to pursue APOE genotyping.

If patients, care partners, or referring clinicians request APOE genotyping prior to the decision to use aducanumab or if the individual has determined their genotype through a commercial service, the Expert Panel recommends that the clinician be prepared to discuss the increased risk for ARIA in the presence of an APOE-4 allele as well as the consequences, monitoring, and management of ARIA if it occurs (discussed below). Genotyping provides transgenerational information on risk of AD for first degree relatives. Parents, siblings, and children of APOE-4 heterozygotes have a 50% chance of being an APOE-4 carrier with an increased risk of AD, and first-degree relatives of APOE-4 homozygotes have a 100% chance of being APOE-4 carriers and have an increased risk of AD. Clinicians may request genetic counseling to assist patients and caregivers in understanding the implications of their genotype (37, 38).

#### Neurological, medical, and psychiatric illness

The Expert Panel recommends that patients with neurological disorders that could account for or contribute to the clinical syndrome of the patients not be treated with aducanumab. This would include patients with parkinsonism, evidence of stroke or widespread white matter ischemic changes, or rapidly progressive dementia. Similarly, recent major psychiatric illness may compromise the ability to adhere to therapy and treatment should be deferred until behavioral stability is established. Poorly controlled or serious medical illnesses (e.g., cancer, heart failure) were exclusions for trial participation and if such illnesses are present in an individual being considered for treatment with aducanumab, the medical condition should be managed and stable prior to initiating treatment. Exclusionary factors are often less rigorous in routine care than in clinical trials but should not be so different as to threaten the generalizability of the trial results to the patient or increase the risk of treatment (39).

Aducanumab has not been studied for its reproductive or teratogenic effects and aducanumab should be administered to younger sexually active AD patients only if they are using contraceptive methods.

#### Clotting status

Aducanumab is associated with ARIA. Patients with evidence of microhemorrhage on MRI (discussed below) or with clotting abnormalities or who were on anticoagulants were excluded from the pivotal trials. It is not known if these exclusions affected the rate of microhemorrhage associated with aducanumab therapy. The risk of severe ARIA in a person receiving anticoagulants or with a clotting disorder is sufficient to exclude them from treatment with aducanumab. Platelet anti-aggregation agents are allowable as concomitant therapy. Lumbar puncture for confirmation of amyloid status should not be performed on patients being treated with anticoagulants; the occurrence of perispinal hemorrhage and spinal cord compression are low but can occur and the risk should be avoided (40).

#### Concomitant Medications

There are no adverse drug-drug interactions noted in the Prescribing Information (1). Drugs used in routine care of patients with AD were allowed to be used by participants in the pivotal trials. The Expert Panel agreed that aducanumab may be co-administered with other drugs used in the treatment of AD including cholinesterase inhibitors (donepezil, rivastigmine, galantamine), memantine, and psychotropic agents (antidepressants, antipsychotics, hypnotics).

#### MRI prior to initiating treatment

Concern for the occurrence of ARIA motivated avoiding administration of aducanumab to patients who had evidence of substantial cerebrovascular disease at baseline in the pivotal trials. The protocol excluded patients who had acute or subacute hemorrhage, macrohemorrhage, greater than 4 microhemorrhages, cortical infarction (>1.5 cm), 1 lacunar infarction (>1.5 cm), diffuse white matter disease, or any areas of superficial siderosis (41). The Expert Panel recommends that these exclusions be observed in clinical practice when choosing appropriate patients for treatment with aducanumab. An MRI including T1, T2 or fluid attenuated inversion recovery (FLAIR), T2* gradient recalled echo (GRE) sequences or susceptibility weighted imaging (SWI), and diffusion weighted imaging should be obtained within 1 year of initiating treatment with aducanumab (and more recently if there is any evidence of stroke since the last MRI). A 3-Tesla magnet MRI will reveal more microhemorrhages than a 1.5 Tesla magnet device, and SWI sequences will reveal more ARIA than GRE images (42). Changes from a baseline scan is the basis for ARIA-related decision making, and the Expert Panel recommends that practitioners use the same MRI device with the same imaging protocol for a given patient whenever possible to assist in comparing the images. Computerized tomography (CT) does not provide sufficient information to determine risk at baseline or to monitor ARIA; individuals who cannot have an MRI (e.g., have a pacemaker incompatible with MRI, metallic brain vessel aneurysm clip, or metallic object in an eye) should not be treated with aducanumab.

#### Knowledgeable engagement

In the clinical trials of aducanumab, informed consent from the patient and care partner were required for participation. In clinical care, formal informed consent is not required but a similar approach should be used to ensure that the patient and care partner/family member/companion understand the requirements for treatment and the expected outcome of therapy. Patients with early AD have the cognitive capacity to comprehend the possible benefit or harms of aducanumab treatment. Key aspects of informed therapy include discussion of requirements for monthly infusions and periodic MRI and the risk of adverse events including ARIA. The anticipated duration of therapy is indefinite and longer treatment with disease-modifying agents is expected to have greater effects on the disease course (43); the optimal duration of therapy is unknown and it may be possible to reduce the frequency of infusions when amyloid levels have been substantially reduced but this has not yet been determined. Those considering aducanumab therapy should understand that the expected benefit is slowing of cognitive and functional decline; improvement of the current clinical state is not anticipated. Patients should have disease state education regarding the course of AD and the availability of cognitive enhancing agents. Educational programs can improve mood, reduce anxiety, and ameliorate caregiver burden (44). The Expert Panel recommends that appropriate use of aducanumab includes providing information on the requirements for treatment and the expected outcomes, potential risks and side effects, and burdens related to administration and monitoring.

Special efforts are required to engage minority patients and to communicate the need for care and the opportunities for treatment. Minority patients report being “unheard” in medical conversations (45). Historically, use of AD therapies such as cholinesterase inhibitors has been less in African American, Latino, and Asian populations than among White AD patients (46). Addressing concerns about the deleterious effects and stigma of diagnosis and raising awareness of potential benefits of disease identification and treatment may influence the willingness of minority patients to discuss cognitive symptoms with clinicians (47). Minority patients often prefer clinicians who share their language and culture (48). The Expert Panel recommends that clinicians strive to engage diverse patients in diagnosis and treatment discussions with the goal of achieving equity among diverse groups in the use of aducanumab.

### Appropriate Treatment

#### Aducanumab initiation and Titration

**Figure 1:**
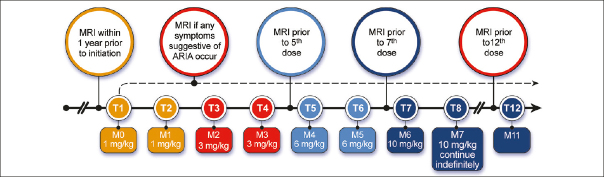
Aducanumab dosing and MRI monitoring schedule (Prescribing Information (1) and Expert Panel recommendation; © J Cummings; illustrator M de la Flor, PhD)

Aducanumab infusions are done monthly and require approximately one hour to complete. Infusions should be at least 21 days apart. The first and 2nd infusion dose is 1 mg/kg; the 3rd and 4th infusions are with doses of 3 mg/kg; the 5th and 6th infusions are dosed at 6 mg/kg; the 7th infusion and beyond involve monthly infusions of 10 mg/kg (Figure 1). Aducanumab is supplied in vials of 170 mg/1.7 mL or 300 mg/3 mL and is added to an infusion bag of 100 mL of 0.9% sodium chloride. The data from the pivotal trials and the Phase 1B trial of aducanumab suggest that 10 mg/kg is the target dose (15). Lower doses may not produce benefit and may cause ARIA. The Expert Panel recommends that patients be titrated to 10 mg/kg. If that is not possible, the clinician should engage in a patient-centered discussion as to whether to continue treatment with lower doses of aducanumab.

Management of missed doses has not been studied. The Expert Panel recommends that if a patient misses a dose, the next infusion should be administered as soon as possible at the dose administered in the previous infusion. If a patient misses three or more doses and requires continued treatment, titration should be re-initiated beginning at a dose level one step below that previously administered (e.g., if the patient was at 6 mg/kg previously, they would resume at a dose level of 3 mg/kg) with the dose increased every other month as described for treatment initiation.

Infusions may be done in a clinician's office; in general infusion centers providing intravenous (IV) therapies to patients with cancer, arthritis, or other disorders; in specialized aducanumab infusion facilities; or at home. Home infusions are administered by a visiting nurse. General infusion center personnel may not be familiar with interacting with cognitively impaired patients and may require specialized training to ensure that the patient has a positive experience fostering a sense of well-being and conducive to treatment adherence. Clinicians should ask patients about any recent symptoms suggestive of ARIA before each infusion. Evidence of coagulopathy, symptoms suggestive of stroke, or poorly controlled blood pressure may be reasons to defer therapy and reevaluate the patient.

#### ARIA monitoring and management

The most common adverse event produced by aducanumab is ARIA. Aducanumab is associated with a substantially increased rate of ARIA compared to rates observed in natural history studies or trial placebo groups. ARIA (ARIA-E and ARIA-H) occurred in 35.2% of patients on high dose aducanumab compared to an occurrence rate of 2.7% in the placebo group (Table 3) (5). Among those receiving aducanumab, ARIA-E was most commonly observed in participants who were APOE-4 gene carriers (43%) and least often in those without the APOE-4 gene (20.3%). Both symptomatic and asymptomatic ARIA are more common in APOE-4 gene carriers. Thirty percent of ARIA-E were mild (< 5cm on FLAIR imaging with hyperintensity confined to one location); 58% were moderate (5–10 cm involving more than one location); and 13% were severe (> 10 cm) (2). Most ARIA occurs in the first 8 months of treatment during the titration period but can occur any time in the treatment course. ARIA was successfully managed in most patients participating in the pivotal trials without discontinuing treatment; ARIA led to discontinuation from the trials in 6.2% of patients on aducanumab and 0.6% of patients on placebo.

**Table 3:** Occurrence of ARIA in the entire population and in participants with and without the APOE-4 allele in the two pivotal trials combined (10 mg/kg dose) (5)

Participant Group	Placebo	Aducanumab
ARIA-E and ARIA-H (overall population)	10%	41%
ARIA-E (overall population)	2.7%	35.2%
ARIA-E with symptoms	10.3%	26%
ARIA-H (overall population)	8.7%	28.3%
ARIA-E APOE-4 carriers	2.2%	43%
ARIA-E APOE-4 noncarriers	3.9%	20.3%
Trial discontinuations due to ARIA	0.6%	6.2%

Most ARIA events (74%) detected by MRI have no accompanying symptoms. Among those with symptomatic ARIA, symptoms were mild in 67.7%, moderate in 28.3%, and severe in 4% (4). The most common symptoms reported were confusion or altered mental status (5%), dizziness (4%), visual disturbances (2%), and nausea (2%) (2). ARIA episodes typically resolved in 4–16 weeks.

MRIs should be obtained at least 1 year prior to the initiation of treatment and more recently (preferably within 6 months) if there is any suggestion of an intervening central nervous system event (e.g., sudden worsening, transient ischemic attacks). After treatment initiation, MRIs should be obtained before the 5th infusion (before initiating the 6 mg/kg dose); prior to the 7th infusion (before infusion of the first dose of 10 mg/kg); and before the 12th infusion (e.g., before the 6th dose of 10 mg/kg). Given the rate of ARIA-E with the 10 mg/kg dose in the phase 3 studies, especially among APOE-4 carriers, some clinicians may decide to obtain an MRI before the 10th dose, after 3 doses of 10 mg/kg have been administered to avoid failure to detect ARIA that may require active management. MRI studies for ARIA should include FLAIR, T2* GRE and quick DWI. An optional 4th sequence would be either 3D T1 or 3D T2 SPACE (depending on the type of MRI available). In addition to these scheduled MRIs, patients should have an MRI whenever they have symptoms suggestive of ARIA such as headache, vomiting and/or nausea, confusion, dizziness, visual disturbance, gait difficulties, loss of coordination, tremor, transient ischemic attack, new onset seizures, or significant and unexpected acute cognitive decline.

**Figure 2:**
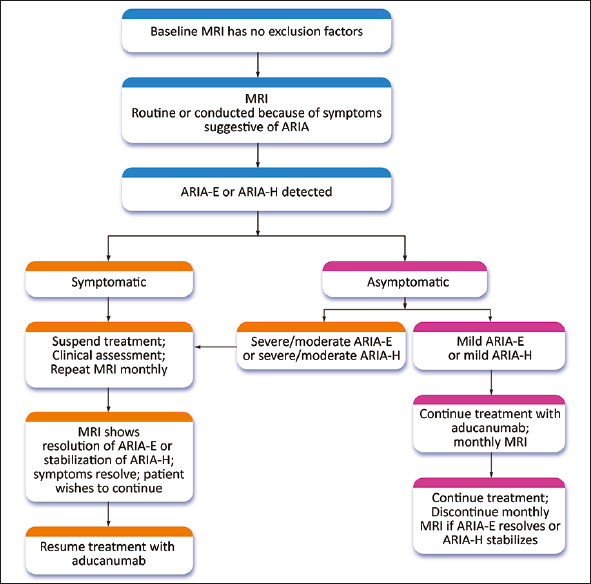
Management strategy for ARIA. Patients with severe symptomatic ARIA are not re-titrated and are not candidates for further treatment with aducanumab (Expert Panel recommendation; © J Cummings; illustrator M de la Flor, PhD)

If patients with ARIA (ARIA-E or ARIA-H) have symptoms, treatment should be suspended, and a clinical assessment and neurological examination performed (Figure 2). MRI should be repeated in 1 month; if the ARIA-E has resolved or the ARIA-H is stabilized, treatment can be resumed. If ARIA-E has not resolved and ARIA-H is worsening, treatment is withheld, and monthly MRIs obtained until treatment can be re-initiated or a decision is made to terminate treatment. If three or more doses are missed before restarting aducanumab, the dose should be re-titrated as described above. Aducanumab should not be re-initiated in patients with severe symptomatic ARIA (e.g., seizure, stroke-like syndromes).

If patients are asymptomatic and the MRI reveals severe or moderate ARIA-E or severe or moderate ARIA-H (Table 4), treatment is suspended, and management follows the procedures described for patients with symptoms (Figure 2). If asymptomatic patients have mild ARIA-E or mild ARIA-H, treatment is continued, and MRIs are obtained at monthly intervals until ARIA-E is resolved or ARIA-H is stable. There is limited information on best practices for management of moderate ARIA-E or moderate ARIA-H and recommendations may evolve.

**Table 4:** MRI severity levels of ARIA-E and ARIA-H as described in the aducanumab Prescribing Information (1)

ARIA-Type	Mild	Moderate	Severe
ARIA-E	FLAIR hyperintensity confined to sulcus and or cortex/subcortical white matter in one location < 5 cm	FLAIR hyperintensity 5 to 10 cm, or more than 1 site of involvement, each measuring < 10 cm	FLAIR hyperintensity measuring > 10 cm, often with significant subcortical white matter / sulcal involvement. May involve one or more separate sites
ARIA-H microhemorrhage	≤ 4 new microhemorrhages	5 to 9 new microhemorrhages	10 or more new microhemorrhages
ARIA-H superficial siderosis	1 focal area of superficial siderosis	2 focal areas of superficial siderosis	> 2 focal areas of superficial siderosis

Clinicians providing aducanumab need access to MRI facilities and to radiologists familiar with detection and reporting of ARIA-E and ARIA-H. Inexperienced readers may fail to detect signs of ARIA when interpreting scans (35, 49). CT is not sufficient for ARIA monitoring.

#### Non-ARIA side effect monitoring

Overall adverse events were experienced by 86.9% of patients on placebo and 91.6% of patients on high dose aducanumab in the pivotal trials (5). Adverse events reported more often in patients receiving aducanumab included headache (20.5% vs 15.2% in placebo), falls (15% vs 11.8% in placebo), and diarrhea (8.9% vs 6.8% in placebo). Serious adverse events occurred in 13.9% of patients on placebo and 13.6% of patients receiving aducanumab. There were 5 fatalities among patients on placebo and 8 among those on aducanumab. The Expert Panel recommends vigilance for all potential side effects in patients treated with aducanumab with special attention to headache, falls, and diarrhea.

#### Effectiveness monitoring

Efficacy was assessed in the pivotal trials using the Clinical Dementia Rating — Sum of Boxes (CDR-sb) (10), Alzheimer's Disease Assessment Scale — Cognitive Subscale (ADAS-cog) (50), Alzheimer's Disease Cooperative Study Activities of Daily Living MCI version (ADCS-ADL-MCI) scale (51), MMSE (11), and the Neuropsychiatric Inventory (NPI) (52). These tools were used to assess patients directly (ADAS-Cog; MMSE; portions of the CDR-sb) or through interviews with care partners (ADCS-ADL-MCI; NPI; parts of the CDR-sb). The time of administration of this panel is approximately 2 hours and some of the instruments take substantial training and experience to be administered reliably (e.g., CDR-sb) (53). Use of such a battery is impractical in many medical or neurological practice settings. Objective assessments requiring less time and training may provide insight in the patient's course; and the clinician should employ tools commonly used in practice. No improvement in cognition or function is anticipated with disease modifying therapy (DMT); slowing of decline and prolongation of the optimal clinical state is the goal of treatment (43). The heterogeneity of decline in early AD makes it difficult to conclude that a slowly progressive disorder is being slowed more by aducanumab (54).

Several means of monitoring treatment effects in the open label practice environment can be considered. The mean change on the MMSE over twelve months in the placebo group in PRIME was (−2.5), in ENGAGE (−3.5), and in EMERGE (−3.3). This provides a range of scores against which the decline in the patient on aducanumab might be compared. The drug-placebo differences observed in EMERGE may guide clinician expectations for the impact of aducanumab on disease progression: this included 18%–27% differences on cognitive decline, 40% difference on functional decline, and 87% difference in behavioral changes. The decline in the late period of MCI due to AD is predictable based on observations in the early MCI period (55). The clinician and care partner may observe differences in the rate of change when aducanumab is introduced and titrated to the 10 mg/kg dose.

The MMSE (11) is commonly used in clinical settings and may be used to monitor patients treated with aducanumab. The MoCA is an alternative to the MMSE (16). The AD8 is a brief informant interview assessing orientation, judgement, memory, and function (56, 57). The AD8 has been shown to have concurrent validity with the CDR used in the pivotal trials and distinguishes patients with MCI (CDR 0.5) from normal elderly with sensitivity of 74% and specificity of 86%. The NPI-Questionnaire is a brief version of the NPI that can be completed by the informant and reviewed by the clinician (58). These three tools are related to or derived from instruments used in the aducanumab pivotal trials. The Functional Activities Questionnaire (FAQ) is a functional rating scale relevant to early AD and is sufficiently brief to be used to assess functional abilities in patients treated with aducanumab (59). The FAQ has good discriminant validity in distinguishing MCI from dementia and performed similarly to the ADCS-ADL-MCI scale in comparative studies (60). These tools are sufficiently brief to be used in practice settings and could be considered for use in evaluating patients receiving aducanumab. Clinicians familiar with CDR administration may consider annual administration of this instrument to assess patient cognitive and functional abilities. The Expert Panel recommends that objective, validated tests to be used longitudinally to assess patients treated with aducanumab.

#### Stopping therapy

The appropriate timing and strategies for stopping aducanumab therapy have not been studied. Stopping treatment might be informed by patient preferences, care partner decisions, or clinician recommendations based on a perceived lack of effect, ARIA-related concerns, or inability of the patient to adhere to the treatment regimen. Aducanumab should be stopped in all patients manifesting severe symptoms (e.g., seizures, stroke-like manifestations) in the presence of ARIA. Stopping treatment in other ARIA-related circumstances depends on whether ARIA-E resolves after suspending therapy, whether ARIA-H stabilizes when treatment is withheld, the patient's clinical status, and clinician-patient alignment on the benefit/harm ratio of resuming treatment.

Aducanumab has not been tested in patients with moderate or severe AD and progression into the more advanced phases of AD will prompt reassessment of treatment continuation. Progression into moderate dementia is signaled by progression to CDR global score of 2.0, decline of MMSE scores below 20, and loss of autonomy on key ADLs. The Expert Panel recommends that clinicians carefully review the evidence of benefit and the potential risk in patients who progress to moderate dementia after appropriate use of aducanumab in early AD.

#### Primary Care Clinicians collaboration

The availability of aducanumab may create a demand for detection, diagnosis and treatment of early AD that can overwhelm an unprepared healthcare system (61). Providing treatment with aducanumab requires high proficiency and sufficient resources including close collaborations with comprehensive multi-disciplinary teams. With too few specialists currently available to respond to the possible number of patients who are candidates for treatment, there are opportunities to forge new models of hub-and-spoke dementia specialist-primary care collaborations and peer-to-peer counseling to partially fill these needs and respond to workforce gaps. The Expert Panel recommends including community organizations, Alzheimer Association chapters, primary care clinicians, memory-care enabled nurses and nurse practitioners, and other creative collaborations and solutions to meet the needs of patients seeking care and encountering the difficulty of being assessed because of shortages of memory care specialists in the current health care system (62, 63, 64).

### Appropriate Patient Discussions

Aducanumab is an unprecedented therapy; it is the first drug approved for treatment of AD based on plaque lowering and addressing the underlying pathophysiology of AD. Clinicians, patients, care partners, and stakeholders of the healthcare system must learn and adjust to the new therapeutic circumstances. Discussions with patients and care partners are particularly important. They require information regarding the possible benefits of aducanumab, the side effects including ARIA, and the likely need for long term adherence to treatment. Dementia medication discontinuation rates have been shown to be higher in African American and Hispanic patients than White patients; racially and ethnically appropriate strategies may be required to optimize adherence (65). Referral to the Alzheimer's Association (www.alz.org) and other trusted community sources can assist the clinician in providing reliable information.

### Aducanumab Treatment in Non-AD Amyloid-Bearing Conditions and Atypical AD

Autosomal dominant AD is produced by mutations of presenilin 1, presenilin 2, or the amyloid precursor protein gene. Patients typically develop amyloid plaques as evidenced by amyloid PET in their mid to late 30's and progress to MCI due to AD and mild AD dementia at age 45 to 55 (66). The individuals have the canonical features of AD at autopsy (67). Few if any of these patients were included in the aducanumab clinical trials. The Expert Panel Recommends that if patients with autosomal dominant AD meet all other criteria for aducanumab treatment described in Table 1, they could be considered candidates for aducanumab and the option can be discussed with families. They should be informed of the scarcity of data in patients with the inherited form of AD.

Individuals with Down syndrome essentially uniformly develop brain amyloid plaques and often have symptoms of dementia in midlife (68, 69). The presence of amyloid plaques in Down syndrome suggests that treatment with aducanumab may be beneficial. There are many differences between Down syndrome and late onset AD, and the Expert Panel recommends against treating Down syndrome patients with aducanumab until more data are available.

Patients with AD may present with atypical syndromes such as logopenic aphasia, posterior cortical atrophy, or frontal AD (70). These patients have metabolic scans that reflect the regional dysfunction corresponding to their clinical presentation; other biomarkers are characteristic of AD (71). Few patients with atypical features were included in the aducanumab trials. The Expert Panel recommends that if patients with atypical AD meet all the criteria for the appropriate use of aducanumab, they can be considered as candidates for aducanumab treatment while cautioning patients and families that little information regarding use of aducanumab is available on patients with these clinical profiles.

Patients with dementia with Lewy bodies (DLB) have MCI that progresses to dementia. They have characteristic clinical features including parkinsonism, visual hallucinations, fluctuating cognition, and rapid eye movement sleep behavior disorder (72). Patients with DLB may have pure Lewy body pathology or may have concomitant Lewy body changes and Aβ plaques. Those with Aβ plaques will have positive amyloid PET imaging (73). The Expert Panel Recommends that patients with DLB not be treated with aducanumab; the effect of treatment in patients with mixed amyloid and Lewy body pathology is unknown.

The ability to image cognitively normal individuals or conduct lumbar puncture and CSF analyses allows the detection of persons in the preclinical phases of the AD continuum. These individuals have amyloid plaques in the brain but are cognitively normal. All participants in the aducanumab clinical trials were symptomatic and met criteria for MCI due to AD or mild AD dementia. There are no data on the utility of treating individuals in the preclinical disease state with aducanumab. The Expert Panel recommends against treating patients in the preclinical phase of AD with aducanumab until additional data are available.

Care partners seek means of improving quality of life for their loved one regardless of the degree of the patient's dementia-related disability. Patients with moderate to severe AD and their caregivers will seek information about aducanumab and may wish to be treated. There are no data available on the use of aducanumab in moderate and severe AD. The Expert Panel recommends against beginning aducanumab therapy in patients with moderate to severe AD (e.g, those with cognitive deficits beyond mild severity and requiring substantial assistance with activities of daily living). These patients require comprehensive compassionate care, and their support must continue regardless of DMT therapy status. Multidisciplinary interventions at this stage can significantly improve quality of life (64, 74).

The amyloid, tau, neurodegeneration (AT(N)) framework is influential in the biomarker classification of AD (75). Using this approach, A+T-N-, A+T+N-, and A+T+N+ patients would be considered candidates for treatment with aducanumab if they have early AD and meet all treatment criteria (Table 1). A+T-N+ patients may have some disorder such as vascular pathology in addition to amyloidosis that may impact aducanumab therapy. Further evaluation of these patients is required before proceeding with therapy.

Patients with cerebral amyloid angiopathy may have positive amyloid PET (76). Use of aducanumab in these patients may promote ARIA (77). The Expert Panel recommends that aducanumab not be used in patients with cerebral amyloid angiopathy.

### Potential Future Changes in Appropriate Use of Aducanumab

AD science is evolving rapidly in both diagnostic and therapeutic technologies. Blood tests that assist in the diagnosis of AD could have a transformative influence on the care of AD patients and the appropriate use of aducanumab. Blood assays that determine the Aß42/40 ratio have good correspondence with amyloid PET status (receiver operator curse area under the curve [AUC] 0.88) and this improves when combined with patient age and APOE-4 genotype (AUC 0.94) (78). Plasma hyperphosphorylated tau (p-tau) 181 and p-tau 217 are abnormal in early AD and correlates significantly with amyloid burden on PET (79, 80, 81). One of these plasma-ased markers or a panel of markers possibly including APOE genotype could eventually provide a diagnosis of brain amyloidosis in patients with symptoms of early AD or could function as a case-finding tool to identify patients likely to have an abnormal amyloid PET.

Blood tests may not be the only means of identifying amyloidosis in patients with the clinical syndrome of early AD. Amyloid is deposited in the retina in AD, and retinal imaging might be another means of detecting central nervous system amyloidosis (82, 83). Digital biomarkers could play a role in case finding or diagnostic confirmation. Voice and language analyses, for example, are promising means of identifying early AD (84, 85).

Currently, aducanumab treatment is administered with the plan of continuing at least until the patient reaches the moderate stage of AD dementia. However, once significant amyloid lowering has been achieved it may be possible to reduce the frequency of infusions. The durability of amyloid lowering was explored in a trial with another amyloid-targeting monoclonal antibody (86) with encouraging results.

Prevention of AD is an important goal of AD research. Trials of aducanumab during the preclinical phases of AD when the brain has high levels of amyloid but cognition remains largely normal may expand the range of individuals appropriate for treatment (87)

Patients with Down syndrome that meet all the other criteria for treatment with aducanumab may become treatment-eligible when additional studies have been conducted and additional data are available (88).

## Summary

Aducanumab is a new treatment for AD. It provides opportunities and challenges for its introduction into the management of AD patients. Aducanumab requires substantial infrastructure for appropriate administration: expert clinicians skilled in recognition of early AD; amyloid PET or lumbar puncture capability; experts in amyloid PET interpretation or CSF analysis: infusion center availability; and access to MRI and experts in recognition and management of ARIA (Table 5). Genetic counseling may be required in some circumstances, and all patients and care partners require education and support. Building this infrastructure for the appropriate use of aducanumab will require time, resources, and creative planning. Appropriate use of aducanumab requires a commitment to patient-centered care and best-practices for the safe delivery of this new treatment.

**Table 5:** Resources needed for the appropriate use of aducanumab (Expert Panel Recommendations)

• Clinicians skilled in the detection and recognition of early AD
• Amyloid PET access or access to individuals with lumbar puncture expertise
• Experts in amyloid PET interpretation or CLIA-certified laboratory available for CSF measurements
• Infusion resources (office/clinic; general infusion center; AD-specific infusion center; home infusion with visiting nurse)
• MRI access
• Experts proficient in recognition of ARIA on MRI
• Experts proficient in clinical recognition and management of ARIA
• Family and patient education and support resources
• Clinicians and staff who deliver culturally competent care
• Genetic counseling available for patients with questions regarding implications of APOE genotyping and interpretation of genetic testing
